# A qualitative study of the impacts of having an infant or young child with achondroplasia on parent well-being

**DOI:** 10.1186/s13023-021-01978-z

**Published:** 2021-08-06

**Authors:** Kathryn M. Pfeiffer, Meryl Brod, Alden Smith, Dorthe Viuff, Sho Ota, R. Will Charlton

**Affiliations:** 1 The Brod Group, 219 Julia Ave., Mill Valley, CA 94941 USA; 2 Ascendis Pharma, Inc., Palo Alto, CA USA; 3 Ascendis Pharma, A/S, Hellerup, Denmark

**Keywords:** Achondroplasia, Caregivers, Children, Emotional well-being, Quality of life, Work

## Abstract

**Background:**

Currently, there is limited research on how having a child diagnosed with achondroplasia affects parents’ lives. The purpose of the study was to investigate the experiences of parents of infants and young children less than two years of age with achondroplasia.

**Methods:**

Concept elicitation interviews were conducted with parents of children less than 2 years of age with achondroplasia in the United States and Spain. Using grounded theory methods modified for health outcomes research, a qualitative analysis of interview transcripts was conducted. Based on the qualitative analysis, a preliminary theoretical model of the experiences of parents of infants and young children with achondroplasia was developed.

**Results:**

Fifteen parents, including 14 mothers and 1 father from 15 unique families, participated in individual or focus group concept elicitation interviews in the US (n = 9) and Spain (n = 6). The qualitative analysis identified four key parent impact domains, which included caretaking responsibilities, impacts on emotional well-being, having worries and concerns about their child, and impacts on daily well-being. Frequently discussed caretaking responsibilities among parents were managing child’s medical care/treatment (93%), obtaining adaptations/items for child (73%), and monitoring child to avoid complications of achondroplasia (67%). Emotional impacts included feeling stressed/overwhelmed (67%), depressed/sad (40%), and anxious/nervous (33%). Worries and concerns included worry/concern about the future (100%), concerns regarding child’s physical health (87%), worry about child’s social well-being (80%), concern for child’s emotional well-being (73%), and worry about child being able to function independently (67%). Daily well-being impacts included family strain (60%), missed work time (47%), and missed/limited social activities (33%). Based on the qualitative findings, a preliminary theoretical model depicting the experiences of parents of infants and young children with achondroplasia was created.

**Conclusions:**

The study sheds light on the range of impacts that parents of infants and young children with achondroplasia may experience, including caretaking responsibilities, impacts on emotional well-being, worries/concerns regarding their child, and impacts on daily well-being. The theoretical model of parent experiences may provide a helpful framework for informing future research and clinical practice.

## Background

Achondroplasia is the most prevalent form of dwarfism and occurs because of a gain-of-function mutation in the *FGFR3* gene, which ultimately affects bone and cartilage growth [1–3]. Clinical features associated with achondroplasia are short stature, disproportionately short legs and arms, macrocephaly, midface hypoplasia, small chest size, abnormal curvature of the spine, short fingers with a trident-shape to the hands, limited elbow extension but hypermobility in the hips and knees, and bowing of the legs [2].

The potential medical complications associated with achondroplasia vary over the life span, but chronic ear infections, sleep apnea, kyphosis, and low muscle tone are common complications of achondroplasia in infants and young children [4, 5]. Infants and young children diagnosed with achondroplasia may also have respiratory issues, conductive hearing loss, hydrocephalus, and foramen magnum compression and are at greater risk of sudden infant death [4, 5]. Guidelines for the clinical management of achondroplasia in infants and children have been well established [1, 2, 5–9]. There is no cure for achondroplasia, but new treatments for children are currently in clinical development [10, 11].

Although the medical impacts of achondroplasia in infants and children are well documented, there is more limited knowledge of nonmedical impacts, including impacts on daily functioning and general well-being [12–15]. Research has shown that infants and young children with achondroplasia frequently have delays in reaching various developmental milestones in the areas of gross motor skills, fine motor skills, communication, and feeding [16, 17]. Gross motor skills, communication, and self-feeding milestones are especially delayed in the first two years of life [17]. For instance, most typically developing children walk independently by the age of 15 months (90th percentile age of achievement), compared to age 26 months for children with achondroplasia [16]. Children with achondroplasia have also been shown to have a lower average quality of life in terms of physical, emotional, social, and school functioning, in comparison with reference population values [18].

Additionally, there is limited knowledge of how having an infant or young child with achondroplasia impacts parents’ day-to-day lives and quality of life, particularly in the first two years of the child’s life. Research has shown that parents of children with complex medical needs have greater caregiving burdens, including time spent on their child’s medical-related care and supervision [19, 20]. Parents of a child diagnosed with a rare condition face additional challenges, including lack of information about the condition, lack of support groups or social support, and the limited knowledge and experience of healthcare providers [21].

There is evidence to suggest that parents must spend more time caring for their young children with achondroplasia. Infants and young children with achondroplasia require frequent medical monitoring and specialist care visits, which add to parent caretaking time and responsibilities [9]. In addition, young children with achondroplasia need greater caregiver assistance with self-care and mobility and increased monitoring compared to children with typical development [22, 23]. Infants and young children with achondroplasia in particular require careful supervision, and precautions must be taken to avoid risks of serious complications [6–9]. For instance, careful head and neck positioning in infant seats and cribs is needed to reduce the risk of foramen magnum compression and sudden infant death.

Research has also suggested that having a child with a complex medical condition has implications for parents’ emotional well-being. The period following a child’s diagnosis with a rare or chronic condition has been described as a critical time for parents in which they frequently experience strong emotions, such as shock, isolation, anger, denial, guilt, and feelings of helplessness [19, 21]. Parents of children with achondroplasia or pseudoachondroplasia have been shown to express differing emotions in the initial period after their child’s diagnosis, including feelings of sadness, worry/anxiety, helplessness, grief, anger, shock, or denial [24, 25]. Beyond the initial period following a child’s diagnosis, studies have also found that parents of children with chronic or rare conditions may have greater stress, depression, anxiety, worries about their child and the future, and feelings of isolation associated with their child’s condition [19, 21, 26]. Consistent with these findings, research has suggested that parents of children with achondroplasia experience impacts on their emotional well-being, such as increased worries and concerns for their child, but less of an impact on their physical health [18].

Parent caregiving burden and well-being are important to consider because of the implications they have for the development and psychosocial well-being of children with chronic illness. Parents of children with a chronic illness have been shown to experience greater parenting stress compared to parents of healthy children, and parenting stress has been associated with poor psychological adjustment in both parents and children with chronic illness [26]. Research has also suggested that parenting behaviors, such as rejection and coercion, negatively affect the physical functioning and well-being of children with chronic physical conditions, while parental warmth is positively associated with child functioning and well-being [27].

To date, there is no in-depth, qualitative study focused on the broad impacts of having an infant or young child with achondroplasia under the age of 2 years on parents’ daily lives and general well-being. Given the special concerns for children with achondroplasia in these early years, including developmental delays, comorbidities, and risks of serious complications, as well as the critical role of parents in ensuring the healthy development and well-being of children with chronic conditions, it is particularly important to understand the experiences of parents of infants and young children with achondroplasia in the first two years of life.

The main objective of the study was to investigate the impacts of having an infant or young child with achondroplasia under the age of 2 years on parent well-being and daily life, including caregiving, emotional and social well-being, family, and work. The study also investigated the physical signs/symptoms or complications associated with achondroplasia that parents observed in their children, which may impact parent experiences. The qualitative analysis of interview data was then used to construct a preliminary theoretical model to illustrate the experiences of parents of infants or young children less than two years of age with achondroplasia.

## Methods

The qualitative study was conducted based on grounded theory methodologies modified for use in health outcomes [28–31]. Concept elicitation (CE) interviews with experts in achondroplasia were conducted to provide clinical and other background knowledge regarding achondroplasia in children, including implications for parents and families. CE interviews were also conducted with parents of infants and young children with achondroplasia aged less than 2 years. CE interview data were analyzed for themes and sub-themes through an iterative process [29]. The qualitative analysis was then used to construct a theoretical model to illustrate the experiences of parents of infants and young children with achondroplasia. Prior to commencement, this study was approved by Copernicus Group Institutional Review Board (IRB), an independent IRB located in Research Triangle Park, North Carolina, United States (US) (Protocol numbers TBG1-18-117 and 20190578).

### Concept elicitation

A targeted literature review and interviews with 6 clinical experts in achondroplasia and 1 leader of an achondroplasia advocacy organization provided clinical knowledge and other relevant information regarding achondroplasia and its impacts on infants, children, and families. Details regarding expert interview methodology and research findings have been published previously [22, 32].

Based on the review of relevant literature and the findings from expert interviews, a semi-structured parent interview guide was created to capture parent experiences related to having an infant or young child with achondroplasia. The questionnaire served as a general guide for the interviews and included broad, open-ended questions regarding impacts on parents’ day-to-day lives, general well-being, work/employment, and family life, as well as parent observations of child signs/symptoms or complications associated with achondroplasia. Follow-up questions and probes were used to solicit additional information as needed. Interviewers generally followed the lead of participants, so interview questions were not always asked in the same way or in the same order.

To be eligible to participate in the study, parents were required to be: (1) at least 18 years of age; (2) able to read, write, and speak English (US participants) or Spanish (for participants in Spain); (3) a parent of a child diagnosed with achondroplasia and less than 2 years of age at the time of interview; and (4) actively involved in the child’s care. Parents were excluded from the study if they had a cognitive impairment or a medical/psychiatric condition that would make it difficult for them to participate in an interview. This study was an extension of a previously published study that included interviews with parents of children with achondroplasia aged 2 to < 18 years of age [22]. Although 6 interviews with parents of children with achondroplasia less than 2 years of age were conducted for the original study, this subsample of n = 6 was not adequate to reach thematic saturation for parent participants in this child age group. Thus, these 6 interviews were excluded from the previously published study and an additional 9 interviews were then conducted and combined with the original 6 interviews to reach a total sample size of n = 15 parent participants for the current study.

To recruit parent participants for the study, a multi-pronged approach was used, including assistance from advocacy organizations, referrals from clinicians, use of a professional market research organization, and “snowball sampling.” Additional details regarding parent recruitment and recruitment targets have been described elsewhere [22]. A brief telephone screener questionnaire was used by the recruiter to determine respondent eligibility prior to participation. Informed consent was given by parents before each interview. As a token of appreciation, a modest honorarium was given to study participants upon completion of the interview.

Parent CE interviews included individual telephone interviews in the US and Spain and one in-person focus group held in Spain. Individual interviews lasted approximately one hour, and the focus group interview was approximately two hours. Verbatim transcripts based on audio recordings were created for each interview to be used in the analysis.

The qualitative analysis was informed by grounded theory methods used in health outcomes studies, in which a theory of parent experiences is developed based on the analysis of parent interview data, while taking clinical/expert knowledge and previous research into account [28, 29]. Dedoose© Version 8.0.35 was used to conduct the qualitative analysis of interview transcripts [33]. The analysis of transcripts was conducted using an iterative process to identify key conceptual themes and subthemes [29]. Prior to the analysis, an initial code list of impacts (concepts) was created. Interview transcripts were then coded for concepts in the order of interview occurrence. Throughout the coding, emerging concepts were incorporated into the coding scheme as needed.

Based on the qualitative analysis of parent and expert CE interview data, a preliminary theoretical model of the experiences of parents of infants and young children with achondroplasia was created. The aim of the theoretical model was to outline the key impacts on parents associated with having an infant/young child with achondroplasia, as well as to distinguish among proximal and more distal impacts and to highlight factors that could potentially modify impacts on parents.

## Results

### Concept elicitation interviews

Fifteen parents of children with achondroplasia less than two years of age participated in CE interviews from 2018 to 2019 in the US (n = 9) and Spain (n = 6). Participants included 14 mothers and 1 father from 15 different families. Fourteen parents participated in individual interviews in the US and Spain, and one parent participated in a focus group interview conducted with parents of children with achondroplasia of varying ages in Spain. Parent CE participants were diverse in terms of their demographic and background characteristics (Table 1). Three parent CE participants (20%) were also diagnosed with achondroplasia. The demographic and health background of the infants/young children of parent interview participants also varied (Table 2).

**Table 1:** Parent concept elicitation participant demographic characteristics

	Spain (*n* = 6)	US (*n* = 9)	Total (*n* = 15)
Age, mean (SD)	34.8 (3.4)	32.1 (5.2)	33.2 (4.6)
(range)	(32–40)	(24–40)	(24–40)
Relationship to child, *n*(%)			
Mother	5 (83)	9 (100)	14 (93)
Father	1 (17)	0	1 (7)
Marital status, *n*(%)			
Married	3 (50)	6 (67)	9 (60)
Divorced	0	2 (22)	2 (13)
Single	2 (33)	1 (11)	3 (20)
No response	1 (17)	0	1 (7)
Race/ethnicity, *n*(%)^a^			
Asian American	–	2 (22)	–
White/Caucasian	–	9 (100)	–
Education, *n*(%)			
High school or equivalent	3 (50)	2 (22)	5 (33)
College degree	2 (33)	6 (67)	8 (53)
Post-graduate school	1 (17)	1 (11)	2 (13)
Work status, *n*(%)			
Full-time	3 (50)	6 (67)	9 (60)
Student	0	1 (11)	1 (7)
Not working (disabled)	0	1 (11)	1 (7)
Not working (other)	3 (50)	1 (11)	4 (27)
Household income, *n*(%)^b^			
< 20,000	0	1 (11)	–
20,001 to 40,000	3 (50)	1 (11)	–
40,001 to 60,000	2 (33)	2 (22)	–
60,001 to 80,000	0	2 (22)	–
80,001 to 100,000	0	1 (11)	–
> 100,000	0	2 (22)	–
Decline to answer	1 (17)	0	–

Percentages may not add to 100 due to rounding. *SD* standard deviation

^a^US only; response categories are not mutually exclusive, so percentages do not add to 100

^b^Household income was reported in Euros (€) for participants in Spain and in US dollars ($) for US participants

**Table 2:** Demographic and health characteristics for children of parent concept elicitation participants

	Spain (*n* = 6)	US (*n* = 9)	Total (*n* = 15)
Child age (months)			
Mean(SD)	16.0 (4.7)	12.9 (7.2)	14.1 (6.3)
(Range)	(9–22)	(3–22)	(3–22)
Child gender, *n*(%)			
Female	1 (17)	3 (33)	4 (27)
Male	5 (83)	6 (67)	11 (73)
Child’s race/ethnicity, *n*(%)^a^			
Asian-American	–	2 (22)	–
Black/African-American	–	1 (11)	–
White/Caucasian	–	9 (100)	–
Age/time diagnosed with achondroplasia, *n*(%)			
In utero	3 (50)	2 (22)	5 (33)
At birth	1 (17)	3 (33)	4 (27)
< 2 months of age	1 (17)	3 (33)	4 (27)
2–6 months of age	1 (17)	1 (11)	2 (13)
Child has parent(s) with achondroplasia, *n*(%) yes	0	3 (33)	3 (20)
Health status (parent-reported), *n*(%)			
Excellent	0	2 (22)	2 (13)
Very good	2 (33)	1 (11)	3 (20)
Good	3 (50)	3 (33)	6 (40)
Fair	1 (17)	3 (33)	4 (27)
Height (cm)			
Mean (SD)	69.5 (4.0)	64.1 (8.8)	66.2 (7.6)
(Range)	(64.0–76.0)	(45.7–74.9)	(45.7–76.0)
Weight (kg)			
Mean (SD)	8.0 (0.7)	7.7 (2.1)	7.8 (1.7)
(Range)	(7.2–9.0)	(4.6–10.9)	(4.6–10.9)

Percentages may not add to 100 due to rounding. *SD* standard deviation

^a^US only; response categories are not mutually exclusive, so percentages do not add to 100

Ninety-five concepts associated with children’s physical signs/symptoms or complications of achondroplasia (29 concepts) and the impacts of achondroplasia on affected infants/young children and their parents (66 concepts) emerged from the analysis of the parent CE interview data. An analysis of thematic saturation confirmed the adequacy of the sample size to cover the broad range of relevant themes for parents of children with achondroplasia under the age of 2 years. Thematic saturation was assessed for the 15 parent participants in the order of interview occurrence. After the 5th interview, 76% of concepts had been mentioned in the interviews. After the 10th parent interview, thematic saturation was achieved as 96% of concepts were discussed.

Parents noted several impacts associated with having an infant or young child with achondroplasia, including caretaking responsibilities, emotional impacts, worries and concerns, and other daily life impacts in the areas of family, work, and social well-being. Parent reports of caretaking responsibilities associated with achondroplasia are shown in Table 3. The caretaking responsibilities that parents mentioned included having to manage their child’s medical care/treatment (93%, *n* = 14), obtaining adaptations or appropriate items for child (e.g., buying or adapting clothing, toys, or a car seat; 73%, *n* = 11), monitoring child to avoid complications due to achondroplasia (e.g., body positioning, head/neck/back support, breathing; 67%, *n* = 10), extra time caring for child because of achondroplasia (e.g., feeding, carrying; 60%, *n* = 9), advocating for child/educating others about achondroplasia (60%, *n* = 9), and finding childcare or babysitting that meets child's needs (40%, *n* = 6).

**Table 3:** Parent caretaking responsibilities associated with achondroplasia

*n*, % reporting impact/issue	Parent reports by child age					
	< 12 months (*n* = 5)		12 to < 24 months (*n* = 10)		Total (*n* = 15)	
Managing child's medical care/treatment	5	100%	9	90%	14	93%
Obtaining adaptations or appropriate items for child (e.g., buying or adapting clothing, toys, car seat)	5	100%	6	60%	11	73%
Monitoring child to avoid complications from achondroplasia (e.g., body positioning, head/neck/back support, breathing)	5	100%	5	50%	10	67%
Extra time caring for child because of achondroplasia (e.g., feeding, carrying)	3	60%	6	60%	9	60%
Advocating for child/educating others about achondroplasia	3	60%	6	60%	9	60%
Finding childcare/babysitting that meets child's needs	2	40%	4	40%	6	40%

In addition to caretaking responsibilities, parents also discussed the effects of having an infant/young child with achondroplasia on their emotional well-being (Table 4). The most often mentioned emotional impacts on parents were feeling stressed/overwhelmed (67%, *n* = 10), going through an initial period of shock or grief after their child’s diagnosis (60%, *n* = 9), feeling depressed/sad (40%, *n* = 6), and feeling anxious/nervous (33%, *n* = 5). Other emotions parents experienced included feeling grateful (20%, *n* = 3), feeling guilty (13%, *n* = 2), feeling happy/experience of joy (13%, *n* = 2), and having increased empathy (7%, *n* = 1). Parents also discussed a number of coping strategies that they used, including focusing on the positive (20%, *n* = 3), putting or keeping things in perspective (13%, *n* = 2), and desensitizing self (e.g., to hurtful comments from others; 7%, *n* = 1).

**Table 4:** Impacts on parent emotional well-being

*n*, % reporting impact/issue	Parent reports by child age					
	< 12 months (*n* = 5)		12 to < 24 months (*n* = 10)		Total (*n* = 15)	
Feel stressed/overwhelmed	3	60%	7	70%	10	67%
Initial period of shock/grief	4	80%	5	50%	9	60%
Feel depressed/sad	2	40%	4	40%	6	40%
Feel anxious/nervous	2	40%	3	30%	5	33%
Feel grateful	1	20%	2	20%	3	20%
Increased knowledge of achondroplasia	1	20%	2	20%	3	20%
Focus on the positive	1	20%	2	20%	3	20%
Acceptance of child’s condition	1	20%	1	10%	2	13%
Feel guilty	1	20%	1	10%	2	13%
Feel happy/experience joy	0	0%	2	20%	2	13%
Put things in perspective	0	0%	2	20%	2	13%
Increased empathy/understanding	0	0%	1	10%	1	7%
Normalize/achondroplasia is “part of life”	0	0%	1	10%	1	7%
Desensitize (e.g., to hurtful comments of others)	0	0%	1	10%	1	7%

All parents mentioned having worries or concerns for their infant/young child with achondroplasia (Table 5). Parents most frequently indicated having worry or concern about the future (100%, *n* = 15), worry about their child’s physical health (87%, *n* = 13), concern for their child’s social well-being (80%, *n* = 12), worry about their child’s emotional well-being (73%, *n* = 11), and worry about their child being able to function independently (67%, *n* = 10). Some parents expressed having safety concerns related to their child (27%, *n* = 4).

**Table 5:** Parent worries and concerns for their child with achondroplasia

*n*, % reporting impact/issue	Parent reports by child age					
	< 12 months (*n* = 5)		12 to < 24 months (*n* = 10)		Total (*n* = 15)	
Worried/concerned about child’s:						
Future	5	100%	10	100%	15	100%
Physical health	4	80%	9	90%	13	87%
Social well-being	5	100%	7	70%	12	80%
Emotional well-being	2	40%	9	90%	11	73%
Ability to function independently	3	60%	7	70%	10	67%
Safety	0	0%	4	40%	4	27%

Parents also reported impacts in their daily lives related to having an infant or young child with achondroplasia, including impacts on physical health, work, social well-being, and family life (Table 6). In terms of impacts on physical health, some parents indicated feeling tired/exhausted (33%, *n* = 5), and fewer parents mentioned having general health issues (13%, *n* = 2). Parents also discussed work issues related to having an infant/young child with achondroplasia, including missed work time (47%, *n* = 7), changed work schedule (27%, *n* = 4), and leaving a job to care for their child (20%, *n* = 3).

**Table 6:** Impacts on parent daily well-being

*n*, % reporting impact/issue	Parent reports by child age					
	< 12 months (*n* = 5)		12 to < 24 months (*n* = 10)		Total (*n* = 15)	
Impacts on physical health	1	20%	5	50%	6	40%
Tired/exhausted	1	20%	4	40%	5	33%
General health issues	0	0%	2	20%	2	13%
Work/productivity issues	3	60%	7	70%	10	67%
Missed work time	3	60%	4	40%	7	47%
Changed work schedule	2	40%	2	20%	4	27%
Stopped work	0	0%	3	30%	3	20%
Reduced work hours	1	20%	1	10%	2	13%
Social/other impacts						
Friendships/social connections through a community of people with dwarfism	4	80%	5	50%	9	60%
Support from family/friends	3	60%	4	40%	7	47%
Limit social life/activities	2	40%	3	30%	5	33%
Stigma/ignorance	1	20%	3	30%	4	27%
Strained relationships	1	20%	0	0%	1	7%
Impacts on families						
Strain in the family	4	80%	5	50%	9	60%
Increased family closeness	3	60%	4	40%	7	47%
Family travel or vacations	1	20%	4	40%	5	33%
Limit/adapt family activities	1	20%	3	30%	4	27%

The most often noted social impacts on parents were developing new friendships or social connections with others affected by dwarfism, frequently through achondroplasia/dwarfism advocacy organizations (60%, *n* = 9), receiving support from family and/or friends (47%, *n* = 7), limiting their social life or social activities (33%, *n* = 5), and experiencing stigma or ignorance of others (27%, *n* = 4). Impacts on family life discussed by parents included experiencing strain in the family (60%, *n* = 9), increased family closeness (47%, *n* = 7), impacts on family travel/vacations (e.g., planning family vacations around advocacy organization meetings or events; 33%, *n* = 5), and limiting or adapting family activities (27%, *n* = 4).

Finally, parents were asked to report on the physical signs/symptoms or complications that their children experienced due to achondroplasia. The most frequently discussed signs/symptoms or complications that parents observed in their children are shown in Table 7. Clinical features of achondroplasia reported by parents, such as short stature, disproportionately short arms and legs, macrocephaly, kyphosis, and hypotonia, were not included in table reports, as they are considered characteristic of the condition [2]. The most frequently observed physical signs/symptoms or complications associated with achondroplasia in their children included sleep problems (e.g., sleep apnea, snoring; 80%, n = 12), ear problems (e.g., ear infections/fluid in the ear; 60%, n = 9), spinal issues/spinal stenosis (40%, n = 6), trouble breathing while awake (33%, n = 5), speech issues (27%, n = 4), and low stamina/tiring easily (27%, n = 4).

**Table 7:** Physical signs/symptoms or complications of achondroplasia in children

*n*, % reporting sign/symptom or complication	Parent reports by child age					
	< 12 months (*n* = 5)		12 to < 24 months (*n* = 10)		Total (*n* = 15)	
Sleep problems (e.g., sleep apnea, snoring)	4	80%	8	80%	12	80%
Ear infections/fluid in ear	2	40%	7	70%	9	60%
Spinal issues/spinal stenosis	4	80%	2	20%	6	40%
Trouble breathing while awake	2	40%	3	30%	5	33%
Speech issues	1	20%	3	30%	4	27%
Low stamina/tiring easily	0	0%	4	40%	4	27%
Sinus issues/congestion	1	20%	2	20%	3	20%
Acid reflux	3	60%	0	0%	3	20%
Balance issues/falls	0	0%	3	30%	3	20%
Hearing problems/loss	0	0%	2	20%	2	13%
Hydrocephalus	2	40%	0	0%	2	13%
Pain	1	20%	1	10%	2	13%
Underweight	1	20%	1	10%	2	13%
Sweating/hot	1	20%	0	0%	1	7%
Difficulty swallowing	1	20%	0	0%	1	7%
Foramen magnum stenosis	0	0%	1	10%	1	7%

Clinical features of achondroplasia (e.g., short stature, disproportionate shortening of arms/legs, macrocephaly, etc.) discussed by parents are not included in this table

### Preliminary theoretical model

**Fig. 1:**
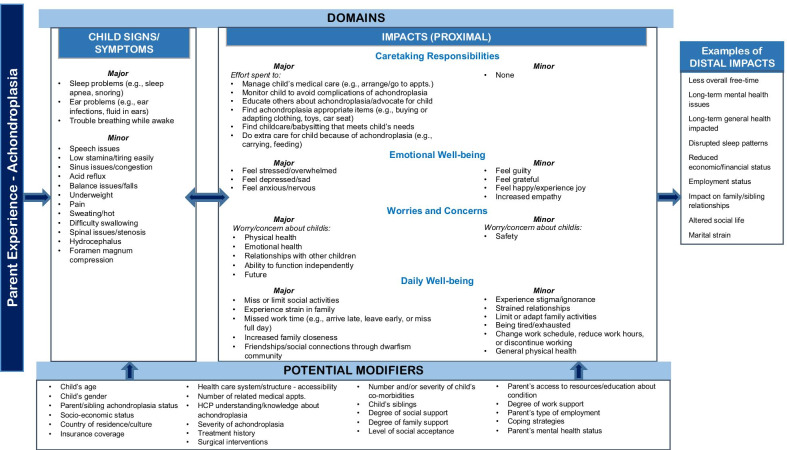
Preliminary theoretical model for parent experiences of having an infant or young child with achondroplasia (ages birth to < 2 years)

Informed by the qualitative analysis of parent interview data, a preliminary theoretical model depicting the experiences of parents of infants/young children under the age of 2 years with achondroplasia was created (Fig. 1). The theoretical model illustrated common signs/symptoms or complications associated with achondroplasia in infants and young children. The preliminary model also identified major and minor impacts on parents in the domains of caretaking responsibilities, emotional well-being, worries and concerns, and daily life impacts (e.g., family life, work, and social well-being). Further, the model distinguished between temporally proximal and distal impacts. Factors that could potentially modify impacts on parents, including child age, severity of achondroplasia, coping strategies, degree of social support, and health insurance coverage, were also noted in the model.

The criteria that the research team used to identify impacts as major were as follows:

1. Endorsement of at least 30% of parents in both child age groups (ages < 12 months and ages 12 to < 24 months)
2. For the child physical signs/symptoms or complications domain, clinical features of the achondroplasia (e.g., small stature, disproportionate shortening of arms and legs, etc. [2]) were excluded
3. Impacts were required to be proximal, rather than distal (temporally)

For the impacts that were not identified as major, the criteria for identifying whether an issue/impact should be designated as minor included:

1. Endorsement from a minimum of 10% of parent participants in at least one of the child age groups analyzed (ages < 12 months and ages 12 to < 24 months)
2. Impacts were required to be proximal, rather than distal (temporally)

Exemplary parent quotes for each of the parent impact domains and major impacts identified are shown in Table 8.

**Table 8:** Exemplary quotes illustrating the impacts of having an infant/young child with achondroplasia on parents

Domain/concept	Exemplary quote
Caretaking responsibilities	
Manage child’s medical care (e.g., arrange/go to appointments)	We have a doctor appointment for him at least every other week, if not every week…there's just a lot more to do…with him being on oxygen, it's a lot harder to go anywhere. There's just so much more to carry with him. (204; parent of 6-month-old)
Monitor child to avoid complications of achondroplasia	…right now, she still doesn't have a 100% head control. So, when the other kids are going down the slide at the park, I don't let her do that by herself…I think she'd fall back because of her head. (248; parent of 22-month-old)
Educate others about achondroplasia/advocate for child	A lot of people just note her size, or they’ll say she’s too young to be doing the things that she’s doing like walking or talking or playing. Not too many people are rude. You can just tell they’re very confused. I just tell them she has dwarfism, achondroplasia, and then they start asking questions… I answer it just to the best of my ability… (211; parent of 21-month-old)
Find achondroplasia appropriate items (e.g., buying or adapting clothing, toys, car seat)	Having special strollers and planning. Most of his stuff we have, it's not custom or anything like that, but having to pick baby gear really specifically to make sure it's safe for his back… (246; parent of 12-month-old)
Find childcare/babysitting that meets child's needs	We went to one [early childcare center] first and it didn't go along with what we want in terms of achondroplasia. They didn't [listen to] us about the recommendations they need to follow, they were not that responsive, and at the end we found the other [childcare] center. From the beginning, they were very responsive… (433; parent of 20-month-old)
Do extra care for child because of achondroplasia (e.g., carrying, feeding)	Well, he can’t dress himself yet…we’re working on [son] feeding himself, because he can’t… with his arms being so short, it’s kind of hard for him…we’re working on getting a spoon from whatever it is he’s eating to his mouth. (224; parent (with achondroplasia) of 19-month-old)
Emotional well-being	
Feel stressed/overwhelmed	I'm super stressed out with all the appointments and going back and forth and seeing different people and making sure that I'm trying to find him the best providers that have experience with achondroplasia. (203; parent of 3-month-old)
Feel depressed/sad	Sometimes, it affects me, in the sense that it makes me feel sad people seeing your son as different. If they knew him, they would realize he is a normal child, he has no problems, it is only at a physical level… It hurts me when people say things about my child, but what can I do? … (434; parent of 22-month-old)
Feel anxious/nervous	I feel a bit anxious about [son] leaving the comfort zone [of family and friends] and going to nursery school or primary school. I know he will adapt whatever the circumstances – he will live with his condition his entire life, but I’m a bit nervous about the social situation anyway… (413; parent of 9-month-old)
Worries and concerns	
Child’s physical health	I feel that it's been—it’s made me very paranoid. I find myself overanalyzing every little thing. If he cries too much then, I'm afraid maybe he's got more fluid on his brain, or there's something going on that I don't know… (203; parent of 3-month-old)
Child’s emotional health	…I worry about her future and how she'll be treated as a teenager and the adolescent years when it's noticeable, and she is aware of how different she is. I do worry about how that will affect her mentally… (248; parent of 22-month-old)
Relationships with other children	I’m worried that he won’t be accepted like everyone else, or that other kids will reject him because he’s different…I’m worried about what he will feel due to the reactions of other kids or parents. You know children can be brutal…They might pick on him more often. (413; parent of 9-month-old)
Child’s ability to function independently	I'm definitely worried about the future all the time and accessibility for him, that's a huge concern. Him being able to be independent is a big worry. Wiping, your own personal hygiene. (246; parent of 12-month-old)
Child’s future	What worries me is that when he is an adolescent, an adult, he is going to have complications…always going to the doctor…when he starts school, we might have to fight for infrastructure for going to the bathroom and all that, he is going to suffer…for something that he did not choose… (431; parent of 15-month-old)
Daily well-being	
Miss or limit social activities	Interviewer: …what about you socially? Are you able to have time to do activities with friends? Parent: Honestly, the first response there was what friends? …I do have a monthly mom group that I go to once a month. So, that's pretty much it. (204; parent of 6-month-old)
Experience strain in family	He really hates the oxygen…It used to disrupt his sleep, but I think that's started to resolve a little bit. Yes. It's been really stressful for our family for him to have to wear it every night… (246; parent of 12-month-old)
Missed work time	Right now, I miss work constantly, but there's a flexibility to make up for the time. If I have to attend any session or appointment, I do it. (433; parent of 20-month-old)
Increased family closeness	I would actually say that it has made family relationship[s] better just between me and the family members. I mean, now, most [of] my family members absolutely love him, aren't afraid of him…I would say a lot of my family kind of overcompensate because of achondroplasia… (204; parent of 6-month-old)
Friendships/social connections through dwarfism community	I'm on four or five different [social media] groups. The Little People of America’s page, the parents of little people page…It's a very, very good support resource, and I've made at least 10 or 15 new friends, at least, online, that I can go to with questions… (204; parent of 6-month-old)

## Discussion

Using rich, qualitative data, this study investigated the broad impacts of having an infant or young child with achondroplasia on parent daily life and general well-being. The findings add to previous research, which has shown that parents of children with chronic or rare conditions often experience stress, anxiety, worries, and sadness/depression and have additional caregiving burdens associated with their child’s condition [19–21, 26]. Parents of infants and young children diagnosed with achondroplasia were shown to have a number of caretaking responsibilities associated with the condition. This finding is consistent with research suggesting that young children with achondroplasia have a greater need for help from caregivers in early childhood [17, 23]. In addition, many parents experienced emotional distress associated with having an infant or young child with achondroplasia, including feelings of stress, depression, and anxiety. Fewer parents reported having physical health issues due to having a child with achondroplasia. These findings are in line with previous research suggesting that parents of children with achondroplasia experienced significantly worse emotional well-being, but no significant difference in physical well-being, in comparisons with a reference population [18].

Parents expressed a wide range of worries and concerns about their child’s functioning and well-being, including worries about the future, concerns about child’s physical health, worries about child’s emotional well-being, concerns regarding child’s social well-being, and worries about child being able to function independently. Parents also discussed positive emotions, such as feeling grateful and having increased knowledge of achondroplasia. Moreover, study results suggest that having an infant or young child with achondroplasia has implications for other areas of parent daily well-being, including strain in the family, limited social activities, and missed work time. Friendships and social support through a community of people affected by achondroplasia, frequently through achondroplasia/dwarfism advocacy organizations, were also important for many parents.

The results also have implications for clinical practice and may be a useful addition to clinical guidelines for the treatment of children with achondroplasia [5–9]. Increased understanding of parent experiences and worries/concerns in children’s first years may facilitate communication and improve support provided to parents and families, particularly for clinicians who may have limited experience with achondroplasia [21]. The evidence affirms the value of considering parents’ emotional well-being, especially in the initial period following a child’s diagnosis [5, 9, 19, 21]. This is particularly important given the association between parent well-being and the healthy development and well-being of children with chronic conditions [26, 27]. In addition to providing parents with education and support resources for achondroplasia, clinicians may also discuss some of the most common worries and concerns that parents expressed, as well as potential impacts on parent caregiving responsibilities and work time. The study findings also support recommendations for parents to connect with advocacy organizations and other parents of children with achondroplasia, which were key sources of education and support for many parents [5, 7, 9, 21].

Study limitations must be considered in the interpretation of results. Given the small sample size, reported percentages and subgroup differences should be interpreted with caution, as they may not be indicative of percentages or group differences found in the study population. Despite the multi-pronged recruitment approach, most parents interviewed were recruited through achondroplasia/dwarfism advocacy organizations. Parents who participate in advocacy organizations may have differing experiences and perspectives compared to parents who are not involved in such organizations. Parents who are involved with advocacy organizations may also be more likely to have personal attributes, such as motivation or self-efficacy, compared to parents who are not involved. Nevertheless, the level and type of engagement with advocacy organizations were found to vary widely among parent participants. For example, some participants only gathered information through an advocacy organization website, while other participants were more actively involved, making social connections, and participating in social activities/events.

Due to the relatively small sample size, it was not possible to explore whether results varied for differing parent demographic/background groups, such as by parent gender, socioeconomic status, or parent knowledge of achondroplasia. Moreover, the extent to which the findings may be generalized to parents of infants/young children with other forms of skeletal dysplasia is unclear, given the numerous types of skeletal dysplasia with differing clinical features, complications, and levels of severity [34]. Further, the results may not be generalizable to other countries, as the experiences of parents may vary in countries with different cultures or healthcare practices. Additional research is needed to examine possible subgroup differences in the findings, as well as the generalizability of results to other populations.

## Conclusions

This is the first study of the broad impacts of having an infant or young child under 2 years of age diagnosed with achondroplasia on parents’ day-to-day lives and parent well-being. The results showed that parent impacts associated with having an infant/young child with achondroplasia included caretaking responsibilities, impacts on emotional well-being, worries and concerns regarding their child, and other daily life impacts related to family, social well-being, and work. The qualitative findings and preliminary theoretical model of parent experiences may inform future research and clinical practice.

## Data Availability

The data for the research presented in this publication may be available from the corresponding author on reasonable request.

## Abbreviations

CE: Concept elicitation
IRB: Institutional Review Board
US: United States
